# Real-time analysis of epithelial-mesenchymal transition using fluorescent single-domain antibodies

**DOI:** 10.1038/srep13402

**Published:** 2015-08-21

**Authors:** Julia Maier, Bjoern Traenkle, Ulrich Rothbauer

**Affiliations:** 1 Pharmaceutical Biotechnology, Eberhard Karls University Tuebingen, Auf der Morgenstelle 8, 72076 Tuebingen, Germany; 2 Natural and Medical Sciences Institute at the University of Tuebingen, Markwiesenstr. 55, 72770 Reutlingen, Germany

## Abstract

Vimentin has become an important biomarker for epithelial-mesenchymal transition (EMT), a highly dynamic cellular process involved in the initiation of metastasis and cancer progression. To date there is no approach available to study endogenous vimentin in a physiological context. Here, we describe the selection and targeted modification of novel single-domain antibodies, so-called nanobodies, to trace vimentin in various cellular assays. Most importantly, we generated vimentin chromobodies by combining the binding moieties of the nanobodies with fluorescent proteins. Following chromobody fluorescence in a cancer-relevant cellular model, we were able for the first time to monitor and quantify dynamic changes of endogenous vimentin upon siRNA-mediated knockdown, induction with TGF-β and modification with Withaferin A by high-content imaging. This versatile approach allows detailed studies of the spatiotemporal organization of vimentin in living cells. It enables the identification of vimentin-modulating compounds, thereby providing the basis to screen for novel therapeutics affecting EMT.

Vimentin, the major intermediate filament of mesenchymal cells, is mainly involved in tissue integrity and cytoarchitecture^1^. The evolutionarily highly conserved protein consists of a central α-helical rod domain, which is flanked by two non-α-helical domains: an amino-terminal head and a carboxy-terminal tail. While the head domain is required for the assembly of vimentin into higher-order filamentous structures, the tail domain is involved in the width control of vimentin filaments^2^,^3^. Assembly and disassembly of vimentin filaments is tightly regulated by the interplay of numerous cellular signaling pathways and modulated by extensive posttranslational modifications^4^.

During the last decade, vimentin has gained much importance regarding its role in key processes of cancer biology, including cell migration and invasion, signal transduction, and apoptosis^5^,^6^,^7^,^8^,^9^,^10^,^11^,^12^. In particular, vimentin has been described as a canonical biomarker for epithelial-mesenchymal transition (EMT), a cellular reprogramming process, in which cells lose their epithelial morphology and acquire a mesenchymal phenotype characterized by a spindle-like shape and increased migratory and invasive properties^13^,^14^,^15^. This process is often accompanied by an extensive upregulation and reorganization of vimentin. In this context, it has been demonstrated that overexpression of vimentin correlates with increased formation of metastases, reduced patient survival and poor prognosis across multiple epithelial cancers, including lung, breast and gastrointestinal tumors^16^,^17^,^18^. The emerging relevance of vimentin in tumor progression turns it into an attractive target for cancer therapy^19^. However, functional elucidation of vimentin in these processes is in an early stage and only few compounds are known that specifically address vimentin as a drug target^11^,^20^,^21^,^22^.

Based on the importance of vimentin as a prognostic biomarker and a molecular target, there is an ongoing demand for novel strategies to study vimentin in disease-relevant models. Currently, most studies rely on antibody-based detection of vimentin in western blot or immunofluorescence. Since such analyses are restricted to endpoint experiments, they do not provide information on dynamic processes. For real-time analysis, microinjection or ectopic expression of fluorescently labeled vimentin has been employed^23^,^24^,^25^. However, steric hindrance affecting posttranslational modification of the head or tail domain cannot be excluded, since the position of the fluorescent moiety is restricted to either the N- or C-terminus of vimentin. Most importantly, ectopic expression of vimentin has been reported to induce changes in cell shape, motility and adhesion and therefore does not reflect the distribution and dynamic organization of endogenous vimentin^26^.

Recently, V_H_H domains (nanobodies, Nbs) derived from heavy-chain-only antibodies of camelids^27^ were fused to fluorescent proteins giving rise to functional fluorescent intrabodies (chromobodies). These chimeric proteins merge the advantages of target-specific binding of antibodies with real-time visualization. Hence, they provide unique information about endogenous protein localization and dynamics in cellular models or whole organisms without affecting protein function and cell viability^28^,^29^,^30^,^31^,^32^,^33^,^34^,^35^.

In this study we developed two vimentin-specific Nbs to follow dynamic changes of endogenous vimentin. We demonstrate that these novel binding molecules are versatile tools to detect vimentin in various biochemical and cellular assays. By generating a bivalent nanobody coupled to an organic dye we established a highly efficient detection reagent for immunoblotting and immunofluorescence studies. For live-cell imaging we introduced vimentin-specific chromobodies into a lung cancer cell model. Following the chromobody signal, we were able for the first time to trace the subcellular localization and redistribution of endogenous vimentin upon siRNA-mediated knockdown, induction with TGF-β and targeted modification with Withaferin A in real time. We monitored and quantified these signal-specific spatiotemporal effects on vimentin in living cells by developing a phenotypic readout based on automated image segmentation for high-content imaging.

## Results

### Identification and generation of vimentin-specific nanobodies

**Figure 1:**
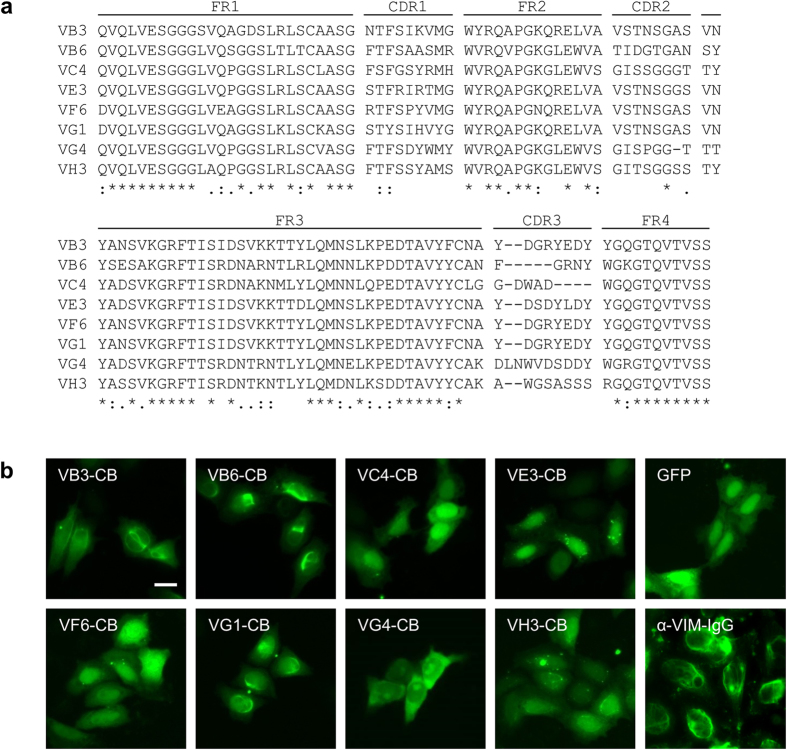
Selection of single-domain antibodies (nano-/chromobodies) against vimentin. (**a**) Amino acid sequence alignment of eight unique vimentin-specific V_H_H/V_H_ domains (nanobodies, Nbs) identified after two rounds of biopanning followed by phage ELISA. (**b**) Representative images of HeLa cells expressing selected sdAbs fused to GFP (vimentin chromobodies). Cells stained with an anti-vimentin antibody (α-VIM-IgG) or expressing GFP served as controls. Scale bar: 20 μm.

To generate vimentin-specific nanobodies, an alpaca (*Vicugna pacos*) was immunized with recombinant vimentin. Subsequently, a phagemid library (~2 × 10^7^ clones) was established from peripheral blood mononuclear cells (PBMCs), representing the respective Nb repertoire. After two cycles of biopanning against full-length vimentin, 47 single clones were analyzed in a solid-phase phage ELISA (Supplementary Fig. 1). Sequencing of the positive clones resulted in eight unique Nb sequences (Fig. 1a). Analysis of the hallmark residues (V_H_/V_H_H: V37Y; G44Q; L45R; W47L) located in framework 2 revealed that four Nbs (VB3, VE3, VG1 and VF6) are derived from heavy-chain-only antibodies, whereas VB6, VC4, VG4 and VH3 are V_H_ domains derived from conventional IgGs^36^. To identify suitable candidates for the envisaged intracellular studies, all selected binders were analyzed in living cells. To this end, we generated chromobody constructs by fusing the coding sequences of the selected Nbs to eGFP and transiently expressed them in HeLa cells. The chromobodies VE3-CB and VH3-CB formed intracellular aggregates, whereas VC4-CB, VF6-CB and VG4-CB were diffusely distributed. Notably, VB3-CB, VB6-CB and VG1-CB displayed a filamentous pattern that resembles the distribution of vimentin (Fig. 1b). Additional structures reminiscent of a midbody pattern were observed in the majority of VG1-CB expressing cells. Hence, we selected VB3 nanobody/chromobody and VB6 nanobody/chromobody as best candidates for further biochemical and cell-biological studies.

### VB3 and VB6 specifically recognize endogenous vimentin

**Figure 2:**
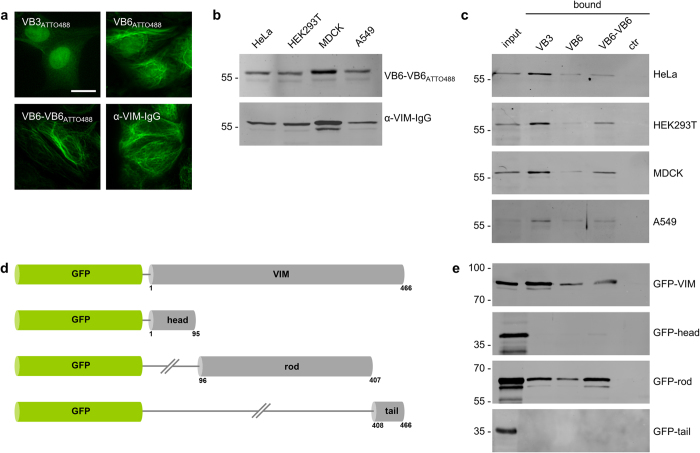
Nanobodies specifically recognize vimentin. (**a**) Immunofluorescence staining of endogenous vimentin in fixed MDCK cells with VB3_ATTO488_, VB6_ATTO488_ and VB6-VB6_ATTO488_ in comparison to α-VIM-IgG. Shown are respresentative images from three independent experiments. Scale bar: 20 μm. (**b**) Immunoblot analysis of protein lysates derived from HeLa, HEK293T, MDCK or A549 cells using VB6-VB6_ATTO488_ for direct detection or an α-VIM-IgG as control. (**c**) Immunoprecipitation of endogenous vimentin with immobilized Nbs. Input and bound fractions of indicated cell lysates were analyzed by immunoblot with an α-VIM-IgG. ctr: pulldown with non-related nanobody. (**d**,**e**) VB3 and VB6 bind to the rod domain of vimentin (**d**) Schematic overview of vimentin domains fused to GFP. (**e**) HEK293T cells were transfected with the indicated constructs and subjected to immunoprecipitation with immobilized Nbs followed by immunoblot analysis of input and bound fractions with an anti-GFP antibody. ctr: pulldown with non-related nanobody.

For *in vitro* analyses, the Nbs VB3 and VB6 were recombinantly expressed and purified from *Escherichia coli (E.coli)*. To detect vimentin by direct immunofluorescence, we chemically coupled an organic dye (ATTO488) to the purified Nbs resulting in a DOL (degree of labeling) of 1 for VB3 and 0.8 for VB6. Staining of fixed MDCK cells with ATTO488-labeled Nbs displayed considerable differences. While VB3_ATTO488_ was primarily localized in the nuclear compartment, VB6_ATTO488_ showed a similar pattern as obtained with a conventional anti-vimentin antibody (α-VIM-IgG) (Fig. 2a upper panel). Since we detected a residual unspecific signal of VB6_ATTO488_, we generated a bivalent VB6 dimer (VB6-VB6). The latter consists of two identical VB6 domains connected by a flexible peptide linker. Cellular imaging with VB6-VB6_ATTO488_ revealed an increase in signal intensity at filamentous structures and a concomitant decrease of unspecific background (Fig. 2a lower panel). In addition, co-staining with VB6-VB6_ATTO488_ and an α-VIM-IgG showed a broad signal overlap at vimentin filaments in HeLa, MDA-MB-231 and MDCK cells while VB3_ATTO488_ and VB6_ATTO488_ only partially colocalized with α-VIM-IgG (Supplementary Fig. 2a–c). Next, we tested whether VB6-VB6_ATTO488_ is also functional in immunoblotting. To this end, 20 μg of soluble protein derived from four cell lines were subjected to SDS-PAGE and immunoblotting. Subsequently, the western blots were stained either with VB6-VB6_ATTO488_ or α-VIM-IgG (Fig. 2b). Direct detection using VB6-VB6_ATTO488_ revealed a high specificity for endogenous vimentin indicated by a comparable band pattern as obtained with α-VIM-IgG detected with a fluorescently labeled secondary antibody (Fig. 2b).

### VB3 and VB6 bind their antigen with high affinities

For antigen production we expressed GFP-labeled vimentin (GFP-VIM) or GFP in HEK293T cells. We calculated the amount of both proteins in the soluble protein fraction by comparing the fluorescence intensities of GFP-VIM or GFP lysates with fluorescence intensities derived from a GFP standard curve (Supplementary Figure 4a,b). For affinity measurements we used biolayer interferometry based on fiber optic sensors with immobilized VB3 and VB6. Incubation with serial dilutions of the soluble protein fraction comprising GFP-VIM revealed K_D_ values of ~11 nM for VB3 and ~37 nM for VB6 (Table 1, Supplementary Figure 4c,d). Notably, only negligible binding was detected in the GFP control lysate (Supplementary Figure 4e).

### VB3 and VB6 precipitate endogenous vimentin

Immobilized nanobodies have been frequently used for immunoprecipitation^34^,^37^,^38^. To test whether the selected Nbs are functional to precipitate endogenous vimentin we incubated the soluble protein fractions, derived from the indicated cell lines, with Nbs covalently coupled to agarose beads. Immunoblot analysis of the bound fractions with an α-VIM-IgG showed that all Nbs bind their antigen but with different efficiencies (Fig. 2c). While only minor amounts of vimentin were detected in the bound fractions of VB6 and VB6-VB6, VB3 efficiently precipitated vimentin.

### VB3 and VB6 bind the central helical rod domain of vimentin

The vimentin monomer consists of three domains: a N-terminal head domain (aa 1–95) containing numerous phosphorylation sites essential for the formation of vimentin filaments^4^, a central helical rod domain (aa 96–407) including the three α-helices (coil 1A, 1B and coil 2), connected by two linkers (L1 and L12) and a C-terminal tail domain (aa 408–488), which is important for the radial compaction of extended filaments^2^,^39^. For domain mapping, we expressed GFP-fusion constructs of the indicated vimentin domains (Fig. 2d) in HEK293T cells and performed immunoprecipitation with immobilized Nbs. The results showed that all Nbs precipitate full-length vimentin as well as the rod domain while none of the Nbs bind to the head or tail domain (Fig. 2e). From these findings we conclude that the epitopes of both Nbs—VB3 and VB6—are located in the rod domain of vimentin.

In summary, our results show that the identified Nbs recognize endogenous vimentin in various biochemical assays. While immobilized VB3 efficiently binds natively folded vimentin in immunoprecipitation, VB6_ATTO488_ can be used for direct immunofluorescence staining of vimentin in fixed cells. Notably, by generating a bivalent VB6-VB6_ATTO488_, we were able to visualize and detect vimentin in immunofluorescence and immunoblotting experiments.

### VB3 and VB6 chromobodies recognize vimentin in living cells

Initially, we observed that the green fluorescent vimentin chromobodies VB3-CB and VB6-CB recognize vimentin-like filamentous structures in HeLa cells (Fig. 1b). Since vimentin binding was confirmed by detailed biochemical analyses, we continued to study the intracellular functionality of both chromobodies more extensively. In a first step, we transiently co-expressed mCherry-tagged vimentin (mCherry-VIM) either with VB3-CB or VB6-CB in HeLa cells (Supplementary Fig. 5a). Imaging of live cells revealed a clear overlap of green and red signals indicating binding of the chromobodies to ectopically expressed vimentin. Additionally, we stained fixed HeLa cells expressing vimentin chromobodies with an α-VIM-IgG. The results show that both, the chromobodies and the conventional antibody, colocalize and recognize endogenous vimentin (Supplementary Fig. 5b).

**Figure 3:**
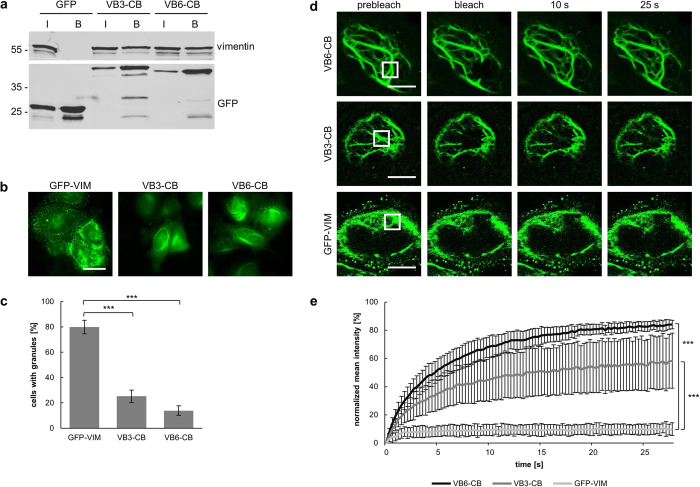
VB3 and VB6 chromobodies recognize vimentin in living cells. (**a**) Intracellular immunoprecipitation (IC-IP) of vimentin. Lysates of HEK293T cells expressing indicated chromobodies (VB3-CB; VB6-CB) or GFP were subjected to immunoprecipitation with the GFP-Trap. Input (I) and bound fractions (B) were analyzed by immunoblot with an α-VIM-IgG (upper panel) and an anti-GFP antibody (lower panel). (**b**,**c**) Vimentin chromobodies have a low tendency to aggregate upon intracellular expression. (**b**) Representative images of HeLa cells expressing GFP-VIM, VB3-CB or VB6-CB from three independent experiments. Scale bar: 20 μm. (**c**) Quantification of cells with fluorescent aggregates upon expression of GFP-VIM, VB3-CB or VB6-CB. Columns represent the percentage of cells displaying fluorescent granules (total number of analyzed cells > 300). Values represent the means of three independent transfections ± stds. For statistical analysis Chi-squared test was used, ***P < 0.001. (**d**) Fluorescent recovery after photobleaching (FRAP) analysis of VB3-CB, VB6-CB and GFP-VIM. Shown are representative images of transiently transfected HeLa cells before and after photobleaching of a defined region (white box). Scale bars: 10 μm. (**e**) Quantitative evaluation of FRAP data showing mean values of fluorescence recovery in photobleached regions. VB6-CB recovered to 84.1 ± 3.1% with a halftime of 3.9 s, the recovery of VB3-CB amounted to 58.4 ± 19.5% with a half time of 4.3 s; n = 10; N = 1. Data are represented as mean ± stds. For statistical analysis students t-test was used, ***P < 0.001.

For further analysis of the intracellular binding properties, we performed intracellular immunoprecipitations (IC-IPs). Soluble protein fractions of HEK293T cells expressing VB3-CB, VB6-CB or GFP as a negative control were subjected to pulldown experiments using the fluorescent moiety as an affinity tag. Input and bound fractions were analyzed by immunoblotting with antibodies against GFP and vimentin. While vimentin was not co-precipitated with GFP alone, it was clearly detected in the bound fraction of both chromobodies (Fig. 3a).

Next, we compared the intracellular distribution of the vimentin chromobodies with GFP-VIM in HeLa cells. Fluorescence imaging showed filamentous structures for all constructs. However, most cells expressing GFP-VIM showed bright fluorescent spots, indicating unspecific accumulation of the overexpressed protein (Fig. 3b). For statistical evaluation, we analyzed the formation of granules in more than 300 cells for each construct. This revealed GFP-VIM aggregates in ~80% of transfected cells. In contrast, the percentage of cells with fluorescent granules was significantly lower upon expression of VB3-CB (~25%) or VB6-CB (~14%) (Fig. 3c). In addition, we investigated whether the expression of the chromobodies affects cell viability. To this end, we performed a resazurin assay 72 h after transfection of VB3-CB and VB6-CB in comparison to GFP (Supplementary Fig. 5c). The results show no significant differences of cellular viabilities in the presence of the chromobodies compared to GFP.

From our previous studies, we know that visualization of intracellular target structures without perturbing their function requires a transient binding mode of chromobodies^29^. To estimate the binding properties of the vimentin chromobodies within living cells, we measured the turnover of vimentin-bound chromobodies by fluorescence recovery after photobleaching (FRAP) analysis. We photobleached the chromobodies or GFP-VIM in HeLa cells and determined fluorescence recovery within the indicated regions (Fig. 3d,e). The results showed that only ~10% of GFP-VIM fluorescence recovered after 25 s. This is in agreement with previous studies describing a slow turnover of fluorescently labeled vimentin filaments^24^,^40^. In contrast, both chromobodies showed significantly higher total recoveries. For VB3-CB we detected a recovery of ~60% and for VB6-CB a recovery of ~85% within the same time frame (Fig. 3e). From these data we conclude that the high recovery rates are not due to the turnover of filamentous vimentin, but rather result from the rapid association/dissociation of both chromobodies. Moreover, compared to VB3-CB (t_1/2_ = 4.3 s), VB6-CB (t_1/2_ = 3.9 s) showed a slightly higher and faster recovery. The differing recovery rates of VB3-CB and VB6-CB are in line with their affinities determined by biolayer interferometry. These results demonstrate that both chromobodies are solubly expressed and recognize endogenous vimentin in living cells without affecting their viability. Based on the FRAP data, we hypothesize that the more transient binding mode of VB6-CB has a minor effect on functionality and dynamic organization of endogenous vimentin and selected this chromobody for further intracellular experiments.

### VB6 chromobody visualizes distribution and reorganization of vimentin

To trace dynamic changes of vimentin in a cancer-relevant cellular model, we chose the lung cancer cell line A549, which has been frequently used to study the EMT process^12^,^41^,^42^,^43^.

Since we observed that transient chromobody expression displays a large intercellular heterogeneity, we developed a set of stable A549 chromobody cell lines by lentiviral transduction of the VB6-CB expression construct. We selected a monoclonal cell line (A549_VB6-CB) with low VB6-CB expression in order to minimize background fluorescence due to an excess of unbound chromobody.

To ensure the validity of the selected monoclonal cell line to study EMT-related processes, we tested whether the genomic integration of the chromobody construct affects the responsiveness to TGF-β stimulation. TGF-β is a widely used EMT inducer, which promotes vimentin expression through Smad-dependent transcriptional activation^44^. First, we analyzed the cellular morphology of wildtype A549 (A549-wt) and A549_VB6-CB cells non-stimulated and stimulated with TGF-β at different plating densities (Supplementary Fig. 6a). The images show no differences in cellular morphology at any of the analyzed conditions. We further characterized the migratory and invasive properties of A549_VB6-CB compared to A549-wt. To that end, we performed wound healing and transwell invasion assays (Supplementary Fig. 6b,c). While the wound healing assay did not reveal any significant differences in migration between the two cell lines, we found that under unstimulated and TGF-β-stimulated conditions the absolute percentage of invading cells of A549_VB6-CB is lower compared to A549-wt. However, the relative increase of invasive cells upon TGF-β stimulation—2.8 fold in A549_wt and 2.9 fold in A549_VB6-CB cells—is virtually identical. Therefore, the two cell lines do not differ in their respective gain in invasiveness upon TGF-β stimulation. In addition, we asked whether the TGF-β-induced change in expression of common EMT marker genes is affected by *VB6-CB* gene insertion. To address this, we performed quantitative real-time PCR (qRT-PCR) and analyzed the mRNA expression of the transcription factors *Snail* (*SNAI1*) and *Slug* (*SNAI2*) and their target gene *E-cadherin* (*CDH1*) relative to *GAPDH* for 0 h, 24 h, 48 h and 72 h of treatment with TGF-β (Supplementary Fig. 6d). In both cell lines the expression of *SNAI1* reached its maximum after 24 h and slightly decreased after 48 h and 72 h, while the expression of *SNAI2* steadily increased over the course of 72 h. In contrast, the mRNA level of *CDH1* was strongly reduced at 24 h and continued to decrease at later time points. Despite differences in the absolute increase of marker gene expression between the two cell lines the overall response to TGF-β is highly similar.

**Figure 4:**
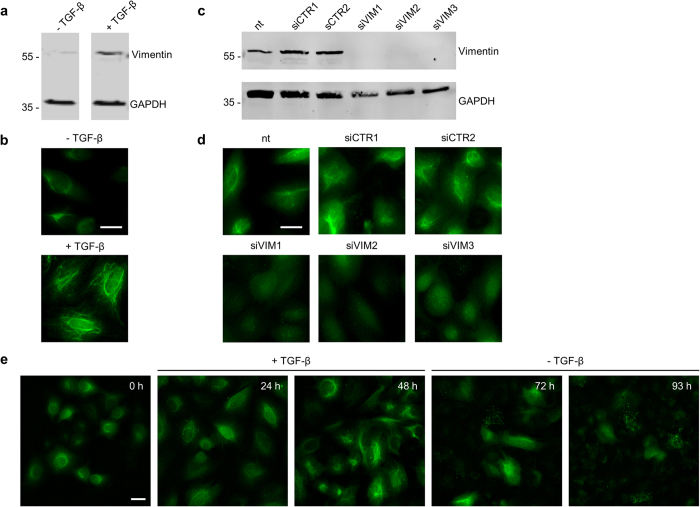
VB6 chromobody visualizes distribution and reorganization of vimentin upon stimulation and knockdown of vimentin. (**a**,**b**) A549_VB6-CB cells were left untreated (−TGF-β) or stimulated with TGF-β (5 ng/ml) for 72 h. Cells were either subjected to immunoblot analysis with an α-VIM-IgG and anti-GAPDH antibody (**a**) or to microscopic analysis (**b**). (**c**,**d**) Knockdown studies of vimentin in A549_VB6-CB cells using three vimentin-specific siRNAs (siVIM1-3) and two control siRNAs (siCTR1-2), followed by immunoblot analysis with an α-VIM-IgG and anti-GAPDH antibody (**c**) or microscopic analysis (**b,d**). Shown are representative images from three independent experiments. Scale bars: 20 μm. (**e**) Time-lapse microscopy of A549_VB6-CB cells. Cells were stimulated with TGF-β (5 ng/ml) for 48 h. Subsequently, TGF-β was removed and cells were cultivated for additional 45 h. Images were taken in 3 h intervals, shown are representative images at indicated time points. Scale bar: 20 μm.

To test whether the chromobody signal is suitable to monitor dynamic modulations of vimentin, we designed two experiments: Firstly, we induced the expression of vimentin by treatment with TGF-β. Secondly, we depleted vimentin by RNA interference. To verify the induction of vimentin, stable A549_VB6-CB cells were either left untreated or incubated with TGF-β for 72 h and corresponding cell lysates were analyzed by immunoblotting. While a minor amount of vimentin was detected in untreated cells, the level was drastically increased upon exposure to TGF-β (Fig. 4a). In parallel, we analyzed the induction of vimentin microscopically. In the absence of TGF-β we observed a weak chromobody signal located mainly around the nucleus. Notably, vimentin filaments extended throughout the entire cell after treatment with TGF-β (Fig. 4b). These findings are in accordance with previous reports describing a TGF-β-induced redistribution of vimentin^45^. Next, we studied the localization of VB6-CB after knockdown of vimentin. To this end, we transfected A549_VB6-CB cells with vimentin-specific siRNAs (siVIM1-3) or two unrelated siRNAs (siCTR1, siCTR2) as controls. Immunoblot analysis after 72 h showed a highly efficient knockdown with all three vimentin-specific siRNAs (Fig. 4c). Accordingly, cellular imaging revealed a diffuse distribution of the chromobody in the absence of its respective antigen (Fig. 4d).

We then asked whether it is possible to trace dynamic changes of vimentin in real time. For time-lapse analysis, we continuously imaged A549_VB6-CB cells over four days. TGF-β was added at the beginning of the time series. After 48 h the cell culture medium was replaced by TGF-β-free medium and image recording was continued for additional 45 h (Fig. 4e, Supplementary movie 1). Images of the time series showed a characteristic redistribution of vimentin filaments originating from the perinuclear region and spreading throughout the entire cytoplasm upon induction with TGF-β. After removal of TGF-β from the medium the reverse process was observed (Fig. 4e). From these observations we conclude that a dynamic redistribution of endogenous vimentin can be readily traced on single cell level following the chromobody signal.

### High-content imaging of the A549_VB6-CB cell model

**Figure 5:**
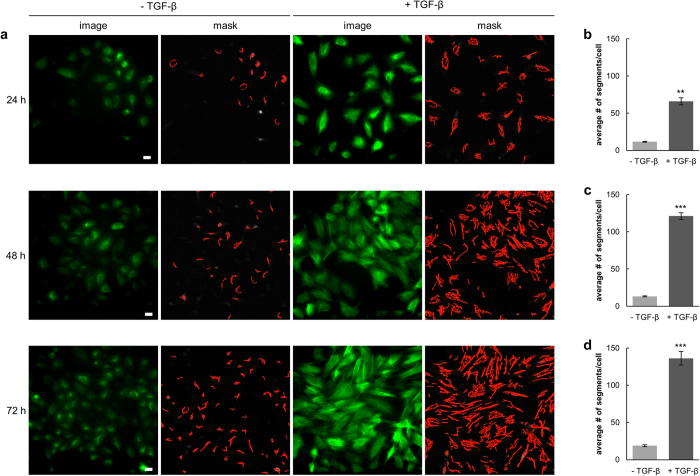
Quantitative analysis of vimentin upon TGF-β treatment. (**a**) Live-cell images of A549_VB6-CB cells left untreated (−TGF-β) or stimulated with TGF-β (5 ng/ml) for the indicated time periods. Shown are raw data images (image) and the respective segmentation of vimentin fibers (mask). Scale bars: 20 μm. (**b**–**d**) Quantification of vimentin fibers in >100 cells after treatment with TGF-β for 24 h (**b**), 48 h (**c**) and 72 h (**d**). Values represent the means ± s.e.m. of three independent experiments. For statistical analysis student’s t-test was used, **P < 0.01, ***P < 0.001.

For high-content imaging and phenotypic screening it is necessary to quantify morphological changes in a statistically relevant number of cells. Therefore, we established a phenotypic readout based on the VB6-CB signal. Within raw data images of A549_VB6-CB cells (Supplementary Fig. 7a) the network of vimentin fibers was automatically recognized and segmented (Supplementary Fig. 7b). Branchpoints were detected to separate vimentin fibers into individual fiber segments (Supplementary Fig. 7b’). In order to calculate the number of fiber segments per cell, nuclei were segmented based on the Hoechst signal (Supplementary Fig. 7c). Thereby, spatiotemporal changes of vimentin structures among different treatments can be compared. We examined this readout in a time-lapse experiment following the induction of vimentin by TGF-β (Fig. 5a). Quantitative analysis revealed a seven-fold increase of fiber segments per cell after 24 h compared to the untreated control (Fig. 5b). Notably, we observed a ten-fold elevation after 48 h, which was not further increased upon longer incubation periods (72 h) (Fig. 5c,d). To demonstrate that our vimentin chromobody model is suitable to monitor the effect of vimentin-modulating small compounds, we performed real-time high-content imaging upon Withaferin A (WFA) treatment. WFA is a steroidal lactone of *Withaferia somnifera* which exhibits anti-tumor and anti-angiogenesis activity *in vivo*^46^,^47^. It has been reported to covalently modify vimentin and thereby to induce dominant negative effects^20^. On molecular level it was shown that WFA promotes phosphorylation and disruption of vimentin^48^. In addition, WFA has been described to inhibit TGF-β-induced EMT in epithelial breast cancer cells^49^.

**Figure 6:**
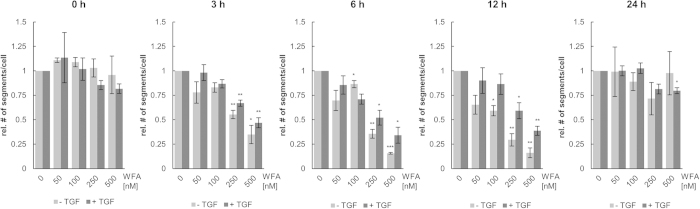
Dose-dependent reduction of vimentin upon Withaferin A treatment. A549_VB6-CB cells were treated with increasing concentrations of Withaferin A (WFA) in absence (−TGF) or presence of TGF-β (+TGF) and subjected to time-lapse microscopy for 24 h. The effect of WFA-treatment on vimentin fiber segments was quantified in >100 cells after 0 h, 3 h, 6 h, 12 h and 24 h. Shown are relative numbers of segments per cell representing the means ± s.e.m. of three independent experiments. Statistical analysis of the dose-dependency was performed using student’s t-test with reference to the untreated control (0 nM WFA), *P < 0.05, **P < 0.01, ***P < 0.001.

To trace the effect of WFA on cells comprising different levels of vimentin, A549_VB6-CB cells were either left untreated or stimulated with TGF-β for 16 h prior to incubation with increasing concentrations of WFA. Subsequently, we monitored the chromobody signal continuously for 24 h. High-content imaging revealed a time and dose-dependent reduction of vimentin fiber segments upon incubation with WFA under both conditions (Fig. 6, Supplementary Fig. 8). Compared to the untreated control (0 nM), incubation with 250 nM and 500 nM WFA led to a significant reduction of vimentin fiber segments after 3 h, 6 h and 12 h (Fig. 6, Supplementary Fig. 8). Moreover, after 6 h and 12 h of WFA treatment, vimentin fiber segments were maximally reduced, whereas after 24 h the number of vimentin fiber segments almost completely recovered to initial levels independently of the WFA concentration (Supplementary Fig. 8). In the presence of TGF-β, treatment with WFA resulted in a two-fold reduction of vimentin within 12 h, while without TGF-β, we observed a ten-fold reduction within the same time period (Fig. 6). Taken together, these data demonstrate that high-content imaging of our vimentin chromobody cell model allows a precise quantification of dose and time-dependent compound effects independently of the initial level of vimentin expression.

## Discussion

Vimentin is the major intermediate filament protein found in mesenchymal cells. Although its role as a component of the cytoskeleton has been known for many years, we are still at the beginning to understand how its dynamic and interdependent functions are orchestrated in a spatial and temporal manner. Hence, novel strategies that enable real-time analysis of vimentin will deepen our insight into the complex regulation of this important mesenchymal biomarker. In this study, we have generated two single domain antibodies VB3 and VB6—referred to as nanobodies, Nbs—to address endogenous vimentin. *In vitro* studies using recombinant VB3 and VB6 coupled to an organic dye have shown that only VB6 detects denatured vimentin in western blot and immunofluorescence. By generating a bivalent VB6-nanobody and thereby increasing its avidity, antigen detection was dramatically improved. Based on these findings, we hypothesize that the VB6 nanobody does not bind a conformational epitope as it is described for most nanobodies^50^,^51^, but rather recognizes a linear amino acid sequence in the rod domain of vimentin. Recently, fluorescently labeled GFP-specific nanobodies have been applied in super-resolution microscopy. Due to their smaller size compared to conventional antibodies, a closer spatial proximity of the fluorophore and the target structure can be achieved^52^. Since fluorescently labeled VB6 directly recognizes vimentin, we assume it to be a promising first candidate to visualize an endogenous antigen in super-resolution microscopy.

During the last years, several studies have shown that single-domain antibodies can be functionally expressed in living cells. Depending on their binding properties they can be either used to visualize or to modulate intracellular target structures^29^,^31^,^32^,^33^,^34^,^35^,^53^,^54^. With VB3-CB and VB6-CB we present two chromobodies as versatile tools to study the localization of endogenous vimentin in live cells. The current state of the art regarding live-cell imaging of vimentin mainly relies on ectopic expression of vimentin fused to fluorescent proteins. However, overexpression of vimentin does not reflect the endogenous situation and can induce dramatic changes regarding cell morphology, migration and adhesion^26^. In contrast, both chromobodies directly bind their endogenous target and visualize vimentin in its physiologically relevant state. This is further supported by a transient binding mode suggesting minimal interference with functions of vimentin. In addition, we speculate that binding of the chromobodies to the rod domain does not perturb posttranslational modification of the N- and C-terminus.

By generating a cell-based chromobody model, we were able for the first time to visualize dynamic changes of endogenous vimentin in real time. Moreover, we developed a high-content imaging approach that allows us to monitor and quantify subtle time and dose-dependent changes in expression and dynamic redistribution of vimentin upon pathway activation^44^, siRNA or compound treatment^20^,^48^,^49^. Current screening strategies to identify novel anti-metastatic compounds affecting EMT are based on endpoint readouts of gene expression, cell growth and migration^55^. As shown in Fig. 6 our proof of principle study demonstrates that our approach to trace an intracellular EMT marker protein provides a much deeper insight into the cellular properties and effects of EMT modulating compounds.

The observation of early effects 3 h after WFA incubation with doses of 250 nM and higher indicates a substantial cellular uptake of WFA and its functionality in the cellular environment. Continuous live-cell imaging revealed a maximum effect after 6 h–12 h and a complete reversion after 24 h which indicates that WFA might be actively exported, inactivated upon metabolization or counteracted by cellular vimentin-regulatory circuits. Such transient compound effects cannot be identified by any endpoint assays. This example clearly illustrates that compound screens which are restricted to certain time points are prone to report false negatives. Our approach is suitable to overcome this limitation and is expected to increase the yield of screening campaigns, thereby facilitating the identification of effective EMT modulators. Additionally, when it comes to downstream preclinical screening including animal studies, such detailed information will support a proper study design (e.g. intervals of compound administration).

In summary our novel chromobody-based approach combines the relevance of vimentin as an essential biomarker for EMT with the unique advantage of live-cell analysis to monitor time and dose dependency of compound-mediated effects. It can be easily adapted to cellular 3D models or even whole organisms and offers a versatile and unique strategy for high-content/throughput identification of anti-metastatic compounds.

## Methods

### sdAb library construction and screening

Alpaca immunizations with purified vimentin and sdAb-library construction were carried out as described previously^29^,^34^. sdAbs with specificity for vimentin were enriched by two consecutive rounds of *in vitro* selection using purified vimentin-His_6_ and individual clones were identified by standard ELISA procedures. For details, see Supplementary methods.

### Plasmids and protein expression/purification

Detailed description of all expression constructs as well as protein expression and purification is provided in the Supplementary methods section.

### Antibodies

The following primary antibodies were used: anti-GFP clone 3H9 (ChromoTek), anti-vimentin, α-VIM-IgG, (R28) (Cell Signaling), anti-vimentin (α-VIM-IgG, clone V9) (Sigma Aldrich), anti-GAPDH (abcam). For detection fluorophore-labeled species-specific secondary antibodies (Alexa-647, Alexa-546, Alexa-488; goat-anti-mouse, goat-anti-rabbit, goat-anti-rat; Life Technologies) were used. Blots were scanned on a Typhoon-Trio laser scanner (GE Healthcare).

### Immobilization of nanobodies on sepharose matrix

1 ml of purified VB3, VB6 and VB6-VB6 at 2 mg/ml in phosphate-buffered saline (PBS) were immobilized on 1 ml NHS-Sepharose (GE-Healthcare) according to the manufacturer’s protocol.

### Labeling of Nanobodies with organic dyes

Purified VB3, VB6 and VB6_VB6 nanobodies were chemically coupled to the organic dye ATTO488 (ATTOTEC) provided as NHS-ester according to manufacturer’s instruction. Unbound dye was removed using PD-10 Desalting Columns (GE Healthcare). Dye labeled protein fractions were analyzed by SDS-PAGE followed by fluorescent scanning on a Typhoon Trio (GE Healthcare, excitation: 488 nM, emission filter settings: 520 nM BP 40). Degree of labeling (DOL, dye-to-protein ratio) was determined by absorption spectroscopy according the instructions provided by ATTOTEC (www. ATTOTEC.com).

### RNAi constructs

For knockdown of endogenous vimentin we used vimentin-specific siRNA duplexes (Life Technologies) with the following sequences (one strand): 5′-GUC UUG ACC UUG AAC GCA Att-3′ (siVIM1), 5′-GGU UGA UAC CC ACU CAA AAtt-3′ (siVIM2), 5′-GAG GGA AAC UAA UCU GGA Utt-3′ (siVIM3). As control siRNAs we used negative control siCTR1 and siCTR2 (Life Technologies).

### SDS PAGE and western blotting

3 × 10^6^ HeLa, MDCK, A549 or HEK293T were seeded in p100 culture dishes and cultivated for 24 h. Cells were washed and harvested in PBS, snap-frozen in liquid nitrogen and stored at −20 °C. Cell pellets were homogenized in 200 μl RIPA buffer (10 mM Tris/Cl pH 7.5, 150 mM NaCl, 0.1% SDS, 1% Triton X-100, 1% Deoxycholate, 5 mM EDTA, 1 μg/ml DNaseI, 2.5 mM MgCl_2_, 2 mM PMSF, 1 × protease inhibitor mix M (Serva)) by repeated pipetting for 40 min on ice and incubated for additional 10 min in a sonication ice bath. After centrifugation (10 min at 18,000 × g) protein concentrations of supernatants were determined by BCA Protein Assay (Thermo Scientific) and adjusted to equal concentrations. Denaturing polyacrylamid gel electrophoresis (SDS-PAGE) was performed according to standard procedures. Protein samples were boiled in 2 × SDS-sample buffer (60 mM Tris/HCl, pH 6.8; 2% (w/v) SDS; 5% (v/v) 2-mercaptoethanol, 10% (v/v) glycerol, 0.02% bromphenole blue). For western blotting proteins were transferred on nitrocellulose membrane (Bio-Rad Laboratories).

### Immunoprecipitation

HeLa, MDCK, A549 or HEK293T were seeded in p100 culture dishes and lysed after 24 h as described above. After centrifugation (10 min at 18,000 × g) the supernatant was adjusted with dilution buffer (10 mM Tris/Cl pH 7.5, 150 mM NaCl, 0.5 mM EDTA, 2 mM PMSF) to 0.5 ml. 10 μl (2%) were added to SDS-containing sample buffer (referred to as input). For immunoprecipitation 30 μl of the agarose-coupled nanobodies were added to the protein solution and incubated for 16 h on an end-over-end rotor at 4 °C. As negative control a non-related nanobody (specific for bovine serum albumin) was used. After centrifugation (2 min, 2500 × g, 4 °C) the supernatant was removed. The bead pellet was washed two times in 0.5 ml dilution buffer, resuspended in 2x SDS-containing sample buffer and boiled for 10 min at 95 °C. Samples (1% input/10% bound) were analyzed by SDS-PAGE followed by western blotting. Immunoblots were probed with anti-vimentin antibody (Cell Signaling).

### Intracellular IP experiments

1 × 10^6^–1 × 10^7^ HEK293T cells were transiently transfected with equal amounts of expression vectors encoding for VB3-CB, VB6-CB (vimentin chromobodies) or eGFP. Transfection efficiency was monitored on the next day by fluorescence microscopy and cells were harvested 24 h after transfection. Cell pellets were lysed as described previously and vimentin chromobodies or eGFP were precipitated using the GFP-Trap (ChromoTek). Input and bound fractions were subjected to SDS-PAGE followed by immunoblotting analysis with anti-vimentin antibody and anti-GFP antibody.

### Immunofluorescence

MDCK, HeLa and MDA-MD-231 were seeded at 8 000 cells per well in μClear 96 well plates (Greiner) and cultured for 24 h. Cells were washed with PBS and fixed with 4% paraformeldehyde (PFA) in PBS. Subsequently, cells were blocked and permeabilized with 3% BSA and 0.1% Triton X-100 in PBS. Staining with primary and secondary antibodies was carried out according to standard procedures. Incubation with Atto488-labeled nanobodies (10 μg/ml in 3% BSA in PBS) was performed for 16 h at 4 °C. For nuclear staining 4′,6-diamidino-2-phenylindole (DAPI, Sigma Aldrich) was used. Images were acquired with a MetaXpress Micro XL system (Molecular Devices) and 40 x magnification.

### Cells culture, transfections and compound treatment

HEK293T, HeLa, A549, MDA-MB-231 and MDCK cells were cultivated according to standard protocols. Briefly, growth media contained DMEM (high glucose, pyruvate) for HEK293T, HeLa, MDA-MB-231 and MDCK cells or DMEM/F-12 (high glucose, pyruvate) for A549 cells supplemented with L-glutamine and antibiotics if not stated differently. Cells were trypsinized for passaging and cultivated at 37 °C in a humidified chamber with a 5% CO_2_ atmosphere. Stable A549_VB6-CB cells were generated as described below. For transient transfection of HEK293T of HeLa cells in p100 dishes, 24 μg DNA were mixed with 140 μL polyethylenimine (PEI, Sigma Aldrich) pre-diluted in 600 μL DMEM to generate DNA/PEI complexes, incubated for 15 min and added to the cells. Transient transfection of A549 cells using Lipofectamine LTX and reverse transfection using RNAiMAX was carried out according to manufacturer’s protocols (Life Technologies). Compound treatment with 5 ng/ml TGF-β (Peprotech) or 50–500 nM Withaferin A (WFA, Merck Millipore) was performed up to 72 h.

### Production of lentivirus and generation of stable cell lines

The VB6 chromobody (VB6-CB) sequence was transferred into pLenti6/V5-DEST by Gateway recombination and lentiviral particles were produced using Virapower lentiviral packaging mix according to manufacturer’s protocols (Life Technologies) with a yield of approximately 5 × 10^7^ transducing units/ml. 24 h after transduction, A549 cells we subjected to a two-week selection period with 80 μg/ml Hygromycin followed by single cell separation in a 96-well plate. Single clones were microscopically analyzed regarding the level of the VB6-CB expression.

### Microscopy and FRAP analysis

Images of the initial selection of the chromobodies and the colocalization studies of the chromobodies and vimentin were acquired with an Axiovert 200M (Zeiss), 40 × magnification. For fluorescence recovery after photobleaching (FRAP) experiments, HeLa cells were seeded in a μ-slide 8-well chamber (Ibidi) and transiently transfected with the plasmids coding for eGFP-Vimentin (GFP-VIM), VB3-CB or VB6-CB. FRAP recordings were performed with a Zeiss confocal laser scanning microscope (CLSM 510 Meta) using a 488 nM Argon laser and 63 × magnification. For photobleaching, the laser was set to 50% output and 100% transmission to bleach a 5 × 5 μm region of interest for 1.7 s. Confocal imaging series were acquired with 1% laser transmission and the pinhole opened to 1.5 Airy units. Generally, 5 prebleach and 145 postbleach images were recorded with 294 ms time intervals. Normalized mean fluorescence intensities were corrected for background and for total loss of fluorescence over time. Fluorescence recovery curves were fitted with Origin 7.5 using an exponential function, given by 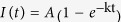, where *I(t)* is the signal intensity dependent on time, *A* is the end value of intensity, *k* is the time constant. Half-times of recovery were determined by 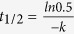. For statistical analysis two-tailed Student’s t-test was used. Phase contrast images for morphological studies of A549 wildtype (A549-WT) and A549-VB6-CB were acquired with a Cell Observer SD (Zeiss) microscope. For time-lapse series of A549_VB6-CB cells were stimulated with TGF-β (5 ng/ml) for 48 h. Subsequently, TGF-β was removed and cells were cultivated for additional 45 h. Images were acquired with an ImageXpress micro XL system, 40 × magnification in 3 h time intervals.

### Image segmentation and analysis

Images were acquired with an ImageXpress micro XL system and analyzed by MetaXpress software (64 bit, 5.1.0.41, Molecular Devices). For automated nuclear segmentation, nuclei of live cells were stained by addition of 2 μg/ml Hoechst33258 (Sigma Aldrich) to the cell culture medium. Automated fiber segmentation, including identification of single segments and branchpoints was carried out based on the VB6-CB fluorescent signal using MetaXpress Custom Module Editor (CME) software (64 bit, 5.1.0.41, Molecular Devices). The total number of fiber segments was divided by the number of segmented nuclei of the entire cell population. For each condition ~300 cells were analyzed. Data derived from WFA experiments were normalized to the untreated control (0 nM WFA). Standard errors were calculated from three independent experiments and Student’s t-test was used for statistical analysis.

## Additional Information

**How to cite this article**: Maier, J. *et al.* Real-time analysis of epithelial-mesenchymal transition using fluorescent single-domain antibodies. *Sci. Rep.* **5**, 13402; doi: 10.1038/srep13402 (2015).

## Supplementary Material

Supplementary Information

Supplementary movie

## Figures and Tables

**Table 1:** Nanobodies bind vimentin with high affinities.

Nbs	K_D_ [nM]	k_on_[1/Ms]	k_off_ [1/s]	R^2^
**VB3**	11.25	6.0 ± 0.11 × 10^5^	6.7 ± 0.16 × 10^−3^	0.98
**VB6**	37	4.75 ± 0.14 × 10^5^	1.76 ± 0.06 × 10^−2^	0.98

The table summarizes affinities (K_D_), association (k_on_) and dissociation constants (k_off_) determined for VB3 and VB6. Corresponding sensograms are shown in Supplementary Figure 4.
