# Supplementary material for: Real-time analysis of epithelial-mesenchymal transition using fluorescent single-domain antibodies
Source: Sci Rep. 2015 Aug 21;5:13402. doi: 10.1038/srep13402 (PMC4544033; doi:10.1038/srep13402)
Supplement: Supplementary Information [file srep13402-s1.docx]

**Real-time analysis of epithelial-mesenchymal transition using fluorescent single domain antibodies**

*Julia Maier^1^, Bjoern Traenkle^1^ and Ulrich Rothbauer^1,2^*

^1^ Pharmaceutical Biotechnology, Eberhard Karls University Tuebingen, Auf der Morgenstelle 8, 72076 Tuebingen, Germany

^2^ Natural and Medical Science Institute at the University of Tuebingen, Markwiesenstr. 55, 72770 Reutlingen, Germany

Correspondence:

Dr. Ulrich Rothbauer, Dept. of Pharmaceutical Biotechnology, University of Tuebingen

Markwiesenstr. 55, 72770 Reutlingen, Germany.

E-mail: [ulrich.rothbauer@uni-tuebingen.de](mailto:ulrich.rothbauer@uni-tuebingen.de)

Phone: +49 7121 51530-415

Fax: +49 7121 51530-816

**Supplementary Information**

**Supplementary Figure 1**

**
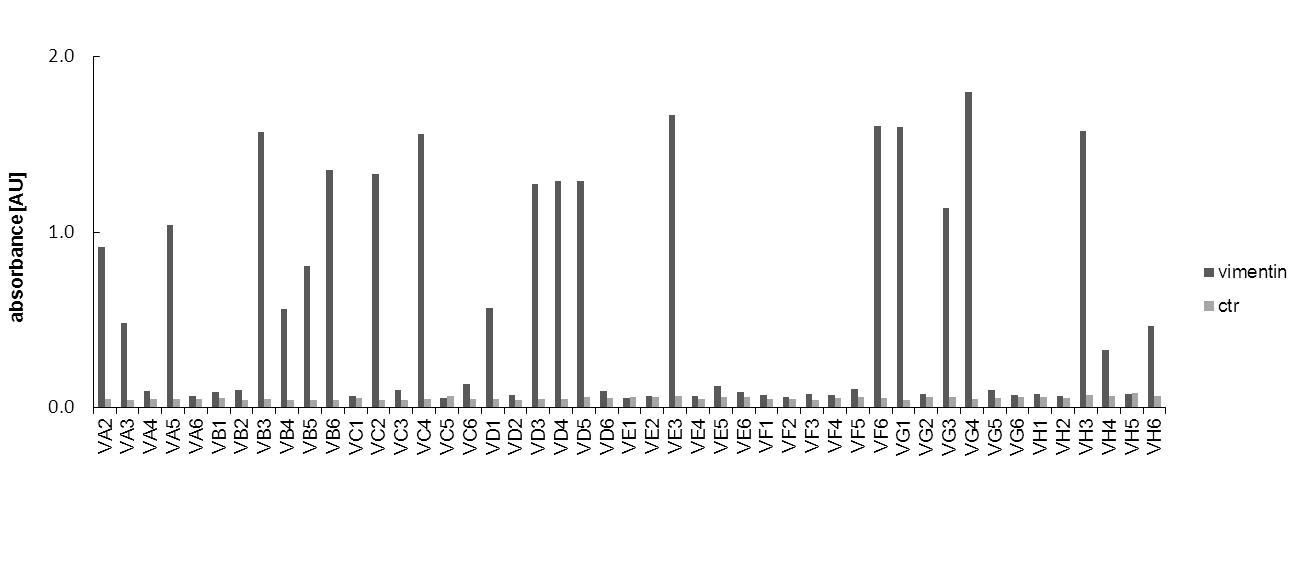
**

**Selection of nanobodies.**

Profile of ELISA measurements on eluted phage clones binding to vimentin. Phage clones comprising vimentin-specific nanobodies were selected by two rounds of biopanning. Phage ELISA was performed with immobilized recombinant vimentin or control protein (ctr: GFP). For detection an HRP-conjugated anti-M13 monoclonal phage antibody was used. Shown is the absorbance measured at 450 nm.

**Supplementary Figure 2**

**
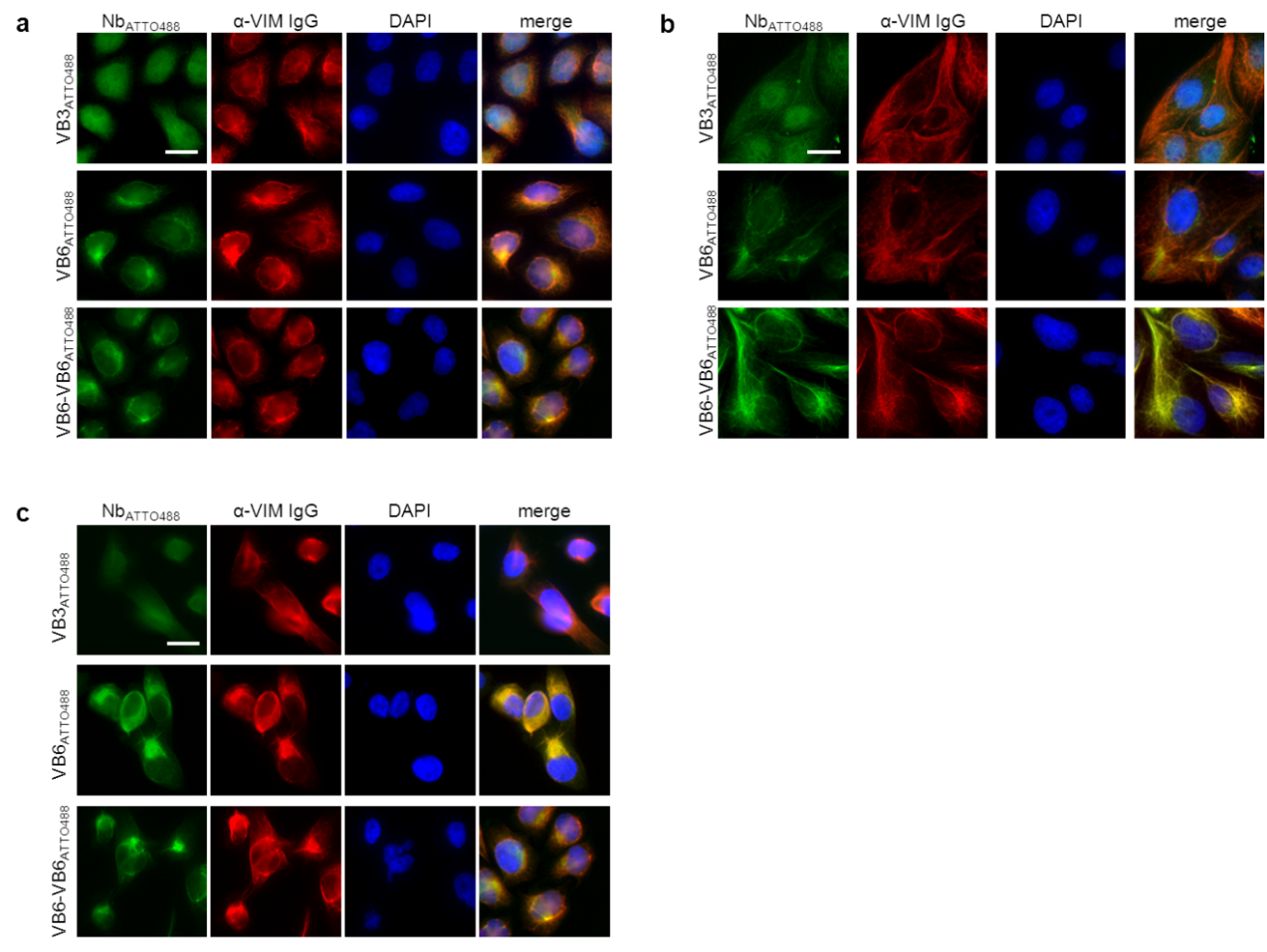
**

**Fluorescently labeled nanobodies recognize endogenous vimentin.**

(**a-c**) Co-staining of fixed HeLa (**a**), MDA-MB-231 (**b**), and MDCK (**c**) cells with VB3_ATTO488_, VB6_ATTO488_ and VB6-VB6_ATTO488_ together with a conventional anti-vimentin antibody (α-VIM-IgG). Shown are representative images from three independent experiments. Scale bars: 20 µm.

**Supplementary Figure 3**

**
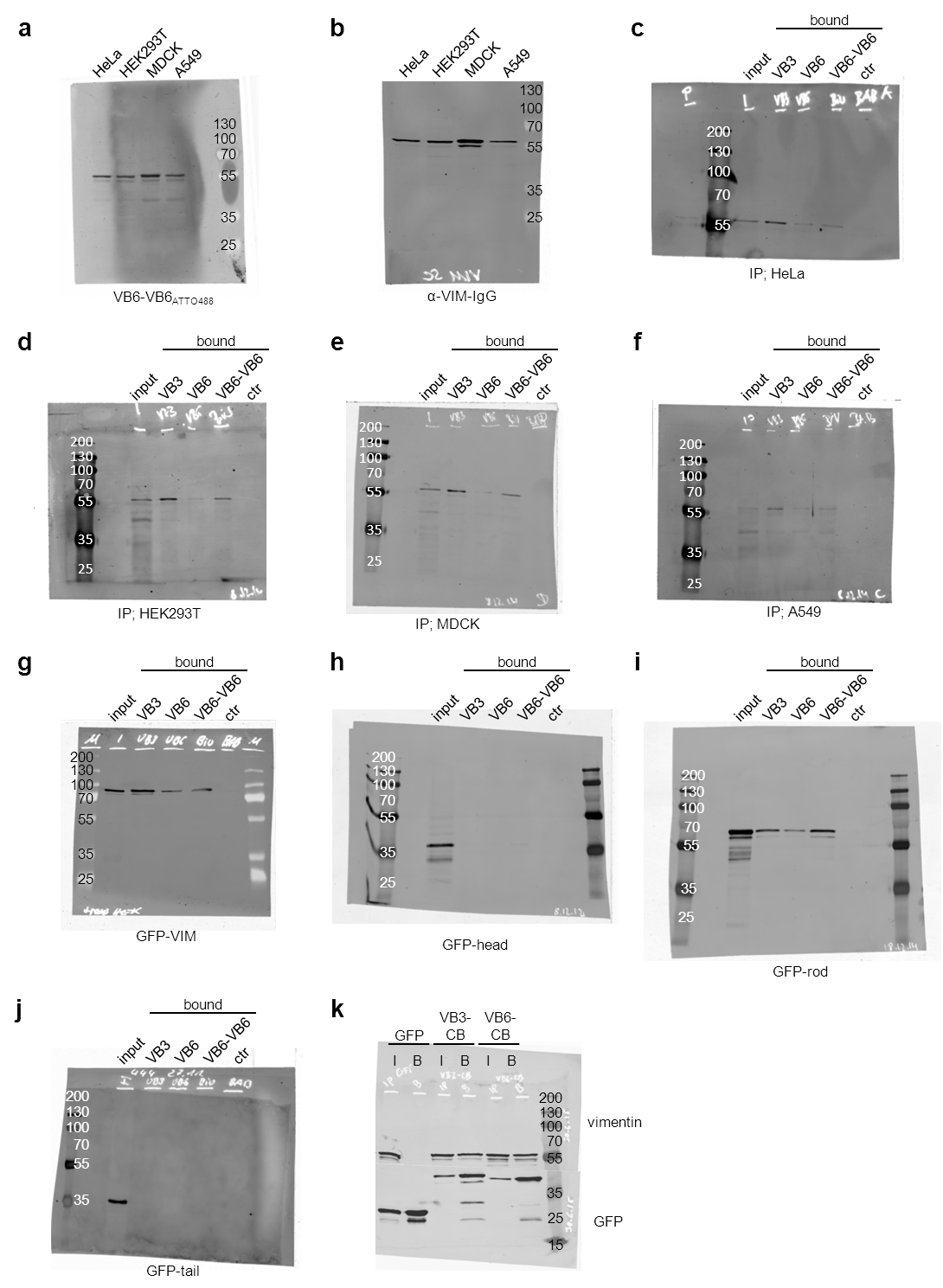
**

**Full western blots** of the experiments shown in (**a**) **Figure 2b** (VB6-VB6_ATTO488_), (**b**) **Figure 2b** (α-VIM-IgG), (**c**) **Figure 2c** (HeLa), (**d**) **Figure 2c** (HEK293T), (**e**) **Figure 2c** (MDCK), (**f**) **Figure 2c** (A549), (**g**) **Figure 2e** (GFP-VIM), (**h**) **Figure 2e** (GFP-head), (**i**) **Figure 2e** (GFP-rod), (**j**) **Figure 2e** (GFP-tail), (**k**) **Figure 4a** (vimentin, upper panel; GFP, lower panel).

**Supplementary Figure 4**

**
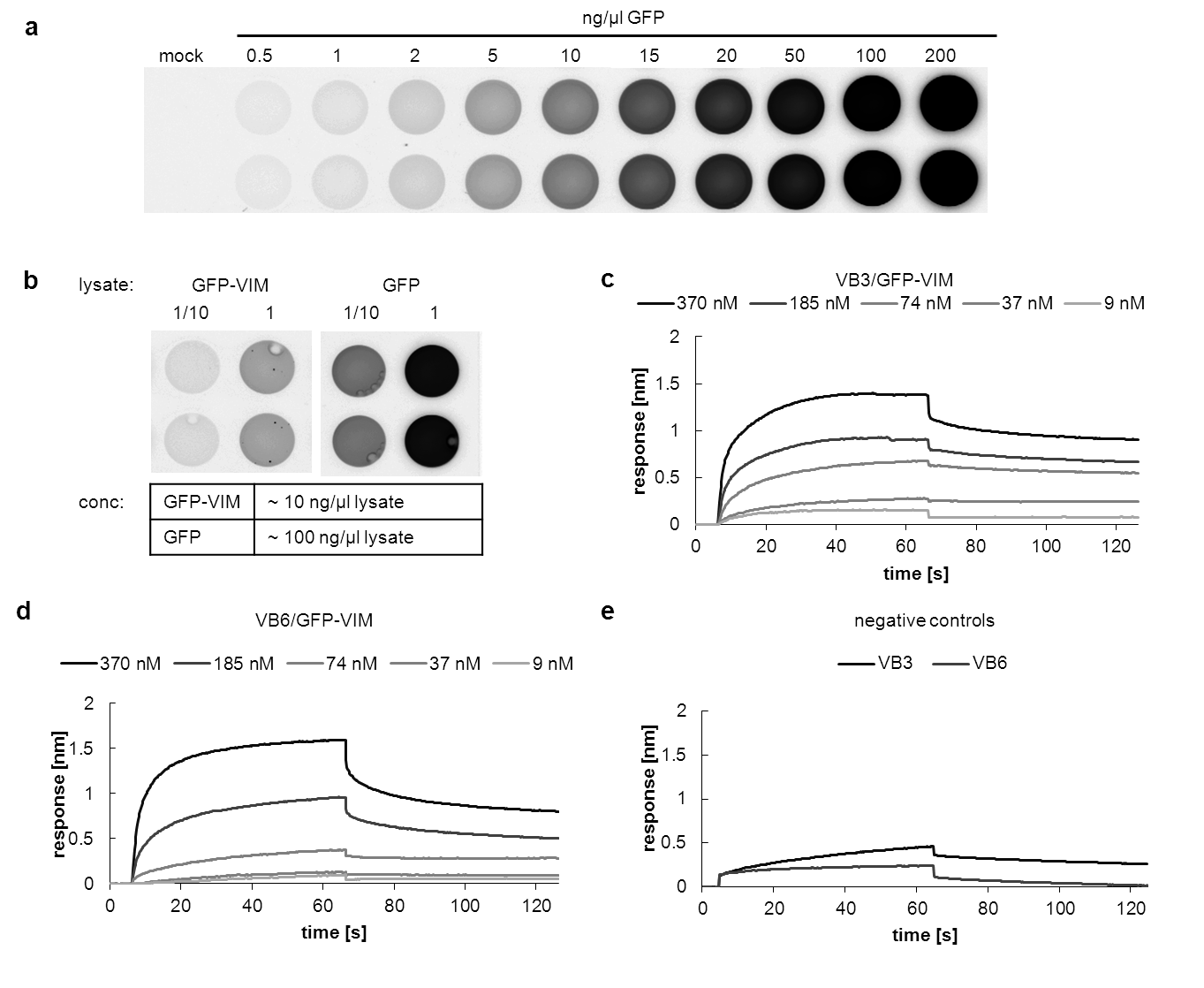
**

**Affinity measurements of vimentin-specific nanobodies.** (**a-b**) Determination of antigen concentration in the soluble lysate fraction. (**a**) Titration of serial dilutions of purified GFP ranging from 0.5 ng/µl (left) – 200 ng/µl (right). The fluorescence signal intensity of GFP was quantified using a laser scanner and translated into a standard curve. (**b**) Aliquots of the soluble lysate derived from HEK293T cells either expressing GFP-Vimentin (GFP-VIM, left panel) or GFP (right panel) were subjected to laser scanning and quantified. Calculated amounts of protein provide the basis for affinity measurements (**c-e**). Binding of Nbs to GFP-VIM or GFP was monitored with the BLItz system which uses fiber optic sensors for detecting protein-protein interactions based on biolayer interferometry (BLI). Purified VB3 (**c**) and VB6 (**d**) nanobodies were immobilized on Anti-Penta-HIS *dip and read* biosensors. Binding affinities were measured by incubation of the Nb-coated biosensors with serial dilutions of the soluble lysate fraction comprising GFP-VIM (9 – 370 nM) followed by a dissociation reaction. The obtained affinities (K_D_), association (k_on_) and dissociation constants (k_off_) determined for VB3 and VB6 are summarized in **Table 1** of the main manuscript. (**e**) Incubation of VB3- and VB6-coated biosensors with soluble lysate fraction comprising GFP (370 nM) served as a negative control.

**Supplementary Figure 5**

**
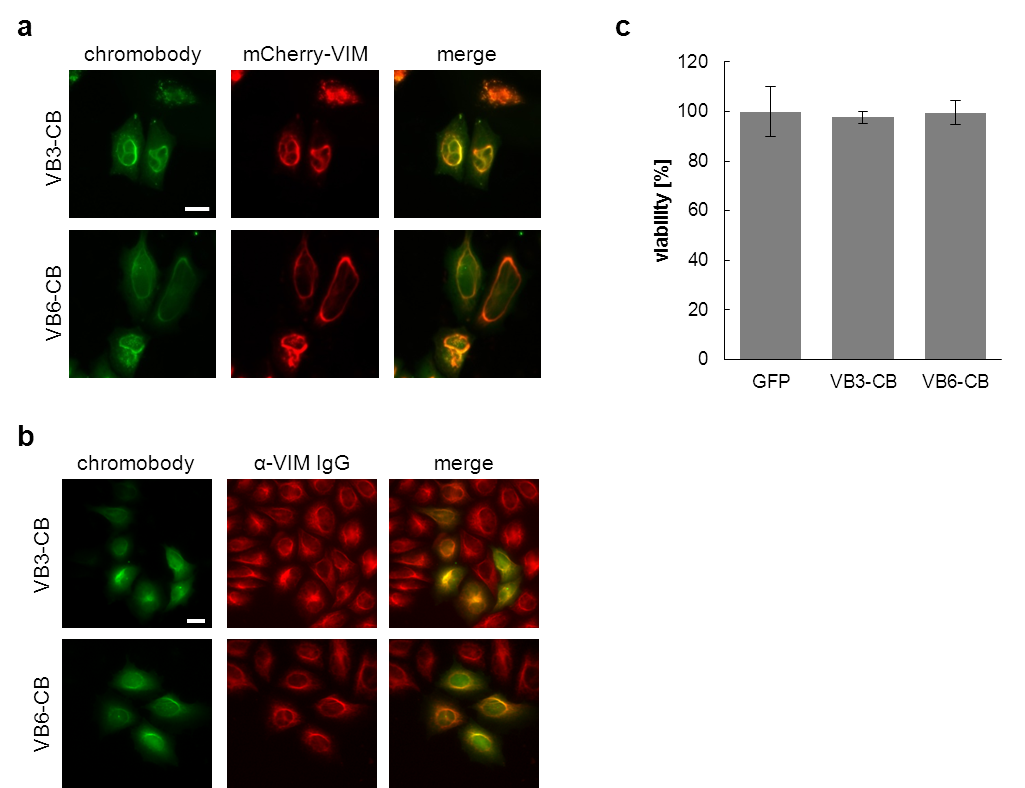
**

**Vimentin chromobodies co-localize with vimentin and are not cytotoxic.**

(**a,b**) Co-localization studies of Vimentin chromobodies. (**a**) Shown are representative images of HeLa cells coexpressing VB3-CB or VB6-CB and mCherry labeled vimentin (mCherry-VIM). Scale bar: 20 µm. (**b**) Representative images of HeLa cells expressing VB3-CB or VB6-CB stained with α-VIM-IgG. Scale bar: 20 µm.

(**c**) Resazurin assay of A549 cells transiently expressing VB3-CB, VB6-CB or GFP. Depicted viability values represent relative mean fluorescence intensities and standard deviation determined for transfection triplicates (± stds) after 72 h. Viability of GFP-transfected cells was set to 100 %. For statistical analysis Student’s t-test was used.

**Supplementary Figure 6**

**
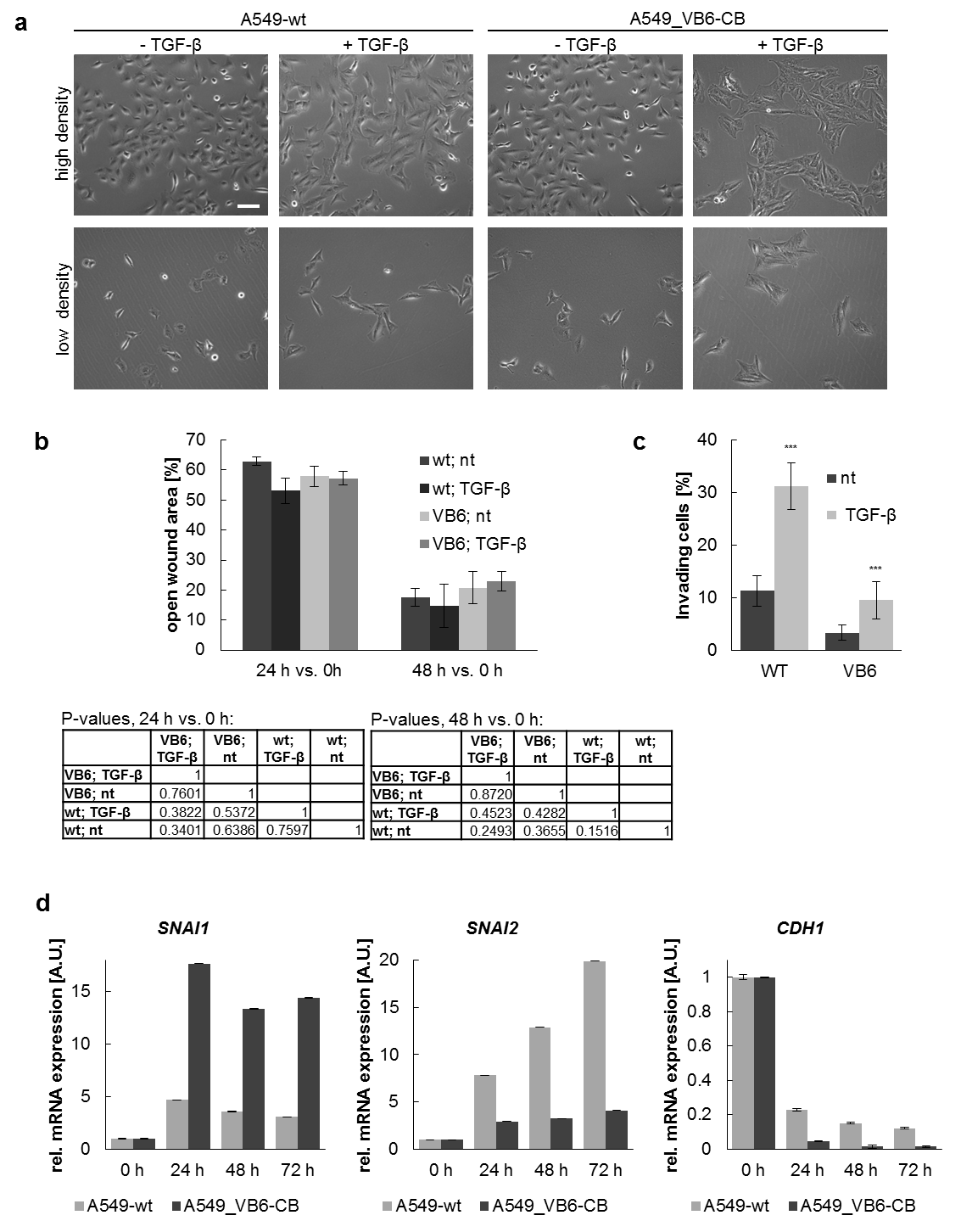
**

**A549-wt and A549_VB6-CB cell lines are highly similar in morphology, migration and gene expression.** (**a**) Representative images of A549 wildtype (A549-wt) and A549_VB6-CB cells either left untreated (- TGF-β) or stimulated with TGF-β (5 ng/ml) for 72 h. Shown are representative images of cells at high (upper panel) or low (lower panel) densities. Scale bar: 100 µm. (**b**) Wound healing assay. A549-wt and A549_VB6-CB cells were grown to 90 % confluence. Scratch wounds were applied and phase contrast images were taken 24 h and 48 h after wounding in the absence or presence of TGF-β (5 ng/ml). Shown are the percentages of the remaining open wound areas of three independent experiments after 24 h and 48 h relative to the 0 h values (24 h vs. 0 h and 48 h vs. 0 h, upper panel). P-values of statistical analysis using student’s t-test are shown (lower panel).

(**c**) Invasion assay. A549-wt and A549_VB6-CB cells were left untreated (nt) or stimulated with TGF-β (5 ng/ml) for 48 h in a matrigel-coated transwell insert. Invading cells were stained with crystal violet. Shown the percentage of invading cells determined with ImageJ n=9, N=1. For statistical analysis student’s t-test was used. (**d**) Analysis of EMT marker gene expression. A549-wt and A549_VB6-CB cells were cultivated in the presence of TGF-β (5 ng/ml) for 0 h, 24 h, 48 h or 72 h. mRNA expression level of *Snail* (*SNAI1*), *Slug* (*SNAI2*) and *E-cadherin* (*CDH1*) was quantified by qRT-PCR in relation to *GAPDH*. Columns represent mean mRNA expression ± stds of three independent replicates.

**Supplementary Figure 7**

**
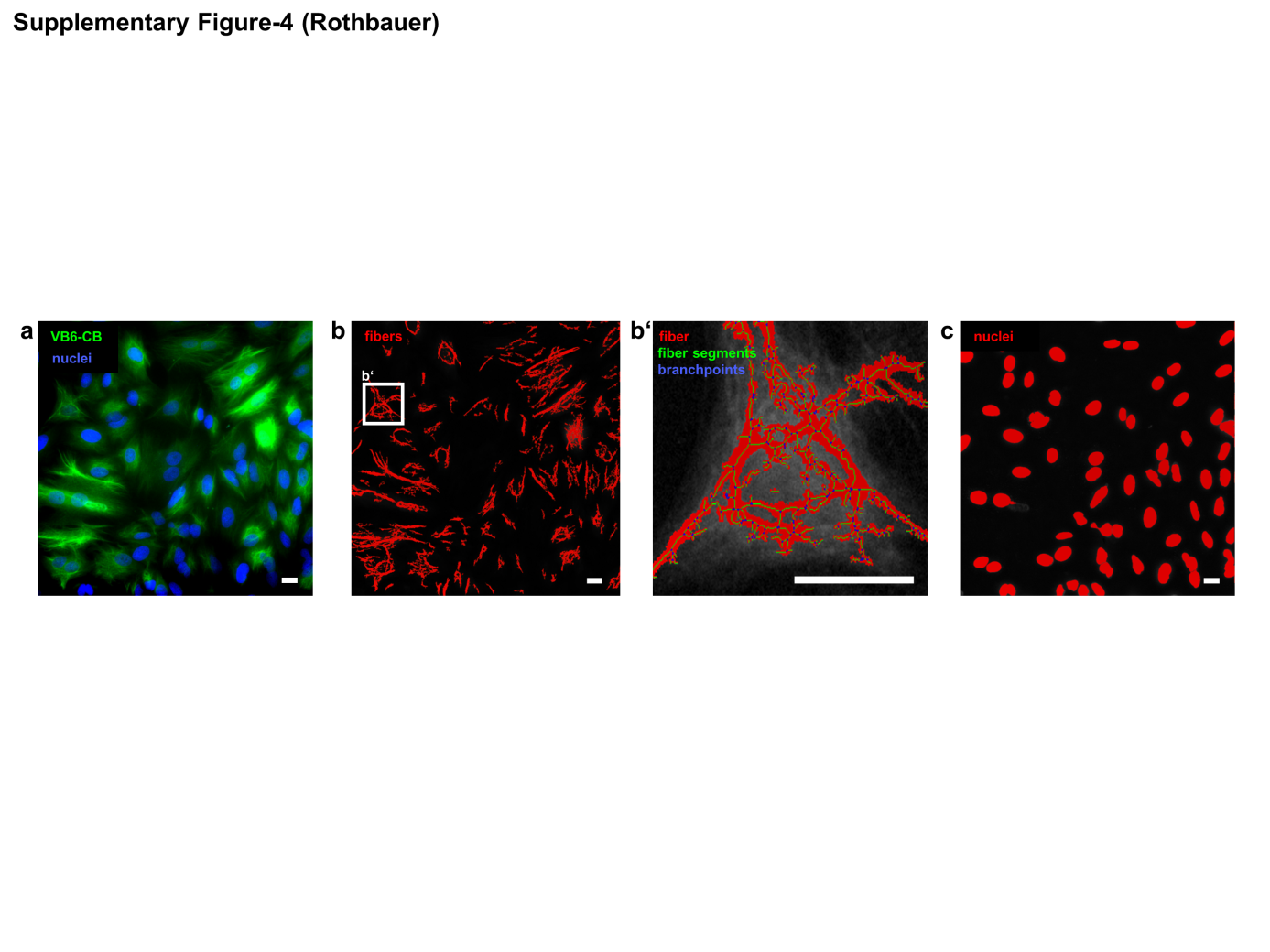
Optical readout based on image segmentation of vimentin filaments.**

(a) Fluorescence image of live A549_VB6-CB cells showing vimentin filaments (green) and nuclear staining with Hoechst (blue). (**b**) Automated segmentation mask of vimentin filaments based on VB6-CB signal using MetaXpress Custom Module Editor (CME) software (64 bit, 5.1.0.41, Molecular Devices). (**b’**) Enlarged segmentation of filament mask (red) including fiber segments (green) and branchpoints (blue). (**c**) Automated segmentation mask of nuclei based on Hoechst signal. Scale bars: 20 µm.

**Supplementary Figure 8**


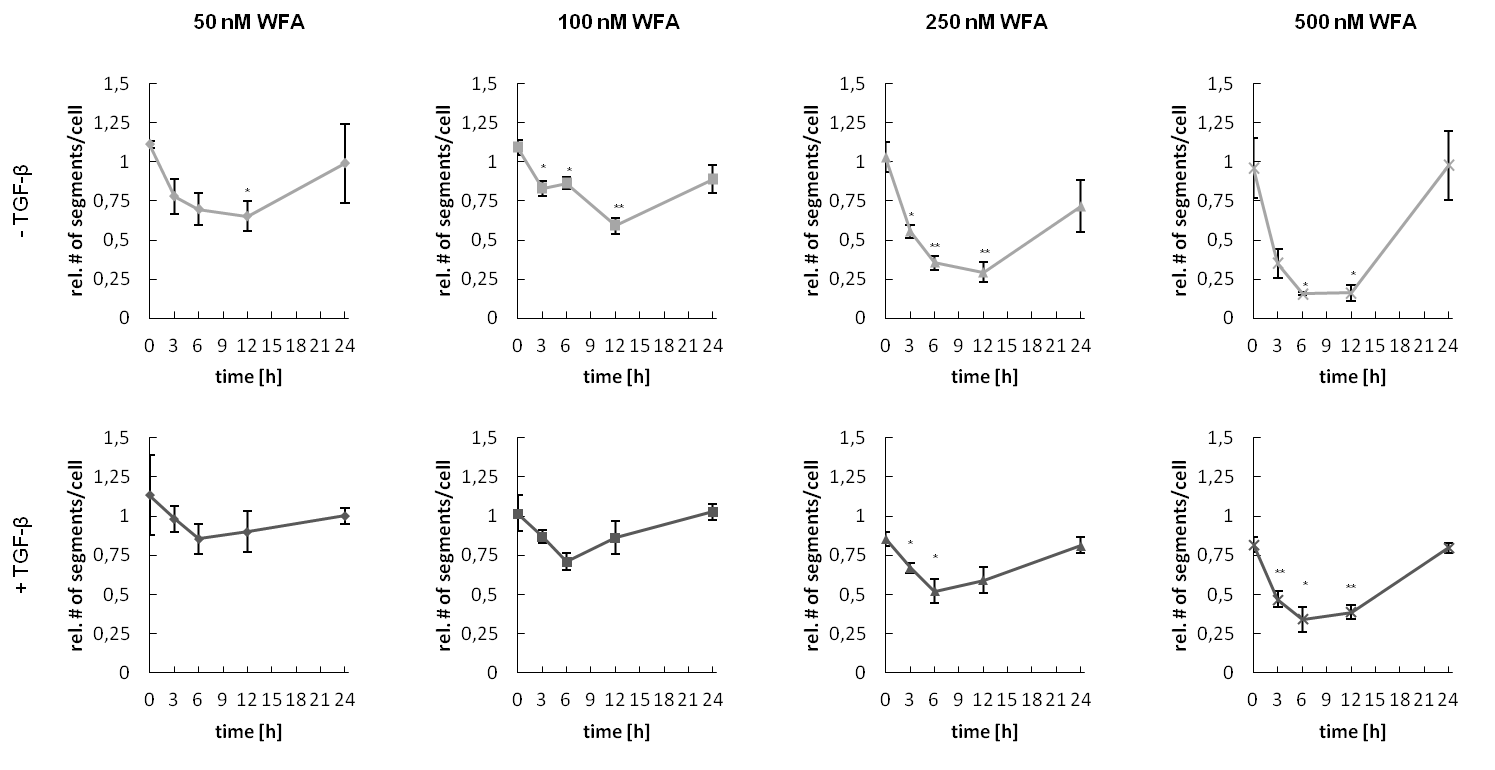


**Time-dependent reduction of vimentin upon Withaferin A treatment.**

A549_VB6-CB cells were treated with increasing concentrations of Withaferin A (WFA) in absence (- TGF) or presence of TGF-β (+ TGF, 5 ng/ml) and subjected to time-lapse microscopy for 24 h. The effect of WFA-treatment on vimentin fiber segments was quantified in >100 cells after 0 h, 3 h, 6 h, 12 h and 24 h. Shown are the identical data sets as in **Figure 6** in the main text, including relative numbers of segments per cell representing the means ± s.e.m. of three independent experiments. Statistical analysis of the time dependency was performed using student’s t-test with reference to the 0 h timepoint, *P<0.05, **P<0.01, ***P<0.001.

**Supplementary Movie**

**Time-lapse imaging of VB6 chromobody.**

Stable A549_VB6-CB cells were stimulated with TGF-β (5 ng/ml) for 48 h. Subsequently, TGF-β was removed and cells were cultivated for additional 45 h. Time-lapse series were performed for 93 h. Images were taken in 3 h intervals. Scale bars: 20 µm.

**Supplementary Methods**

**sdAb libraries.** Alpaca immunizations with purified vimentin protein and sdAb-library construction were carried out as described previously [^1^](#_ENREF_1)^,^[^2^](#_ENREF_2). Animal immunization has been approved by the government of Upper Bavaria (Permit number: 55.2-1-54-2531.6-9-06). In brief, 6 weeks after immunization of two animals (*Vicugna pacos*) with C-terminally histidine (His_6_)-tagged vimentin (vimentin-His_6_), ~100 mL blood was collected and lymphocytes were isolated by Ficoll gradient centrifugation using the Lymphocyte Separation Medium (PAA Laboratories GmbH). Total RNA was extracted using TRIzol (Life Technologies) and mRNA was reverse transcribed to cDNA using a First-Strand cDNA Synthesis Kit (GE Healthcare). The sdAb repertoire was isolated in 3 subsequent PCR reactions using the following primer combinations (1) CALL001 (5´-GTC CTG GCT GCT CTT CTA CA A GG-3´) and CALL002 (5´-GGT ACG TGC TGT TGA ACT GTT CC-3´) (2) SM017 and SM018 (5´-CCA GCC GGC CAT GGC TCA GGT GCA GCT GGT GGA GTC TGG-3´) and (5´-CCA GCC GGC CAT GGC TGA TGT GCA GCT GGT GGA GTC TGG-3´) respectively and reverse primer CALL002 and (3) A4short (5´-CAT GCC ATG ACT CGC GGC CAC GCC GGC CAT GGC-3´) and reverse Primer 38 (5´-GGA CTA GTG CGG CCG CTG GAG ACG GTG ACC TGG GT-3´) introducing SfiI and NotI restriction sites. The sdAb library was subcloned into the SfiI/NotI sites of the pHEN4 phagemid vector ^[3](#_ENREF_3)^.

**sdAb screening.** The sdAb domains were expressed on phages after infecting the cells of the ‘immune’ library in pHEN4 with M13K07 helper phages. sdAb with specificity for vimentin were enriched by two consecutive rounds of *in vitro* selection using purified vimentin-His_6_ coated on microtiter plates (10 µg/well). Bound phages were eluted with 100 mM triethylamine (pH 10.0). The eluate was immediately neutralized with 1 M Tris/HCl (pH 7.4) and used to infect exponentially growing TG1 cells. The enrichment of phage particles carrying the antigen-specific V_H_Hs was monitored by comparing the number of phages eluted from wells with captured versus non-captured antigen. Following biopanning, 47 individual clones of each antigen were screened by phage ELISA procedures using a horseradish peroxidase (HRP)-labeled anti-M13 monoclonal antibody (GE-Healthcare).

**Expression Plasmids.** Vimentin was fused to a C-terminal His_6_-tag using the target backbone vector pRSET-B. Vimentin-coding cDNA was amplified by PCR using the following oligonucleotides: 5’-aag gat cca gcc gcc atg tcc acc agg tcc gtg tc-3’, 5’-aaa agc ttt tag tga tgg tga tgg tga tgt tca agg tca tcg tga tgc-3’. PCR products were purified and digested with BamHI/HindIII for ligation into pRSET-B. For bacterial expression of sdAb, sequences were cloned into the pHEN6 vector ^[3](#_ENREF_3)^, thereby adding a C-terminal His_6_-tag for immobilized metal affinity chromatography (IMAC) purification as described previously [^4^](#_ENREF_4).

For expression of the bivalent VB6-VB6 nanobody a plasmid coding for a bivalent RFP-specific nanobody (RB8) was provided by ChromoTek. The coding sequence of the N-terminal RB8 fragment was replaced by the SfiI/BstEII-digested VB6 fragment. The C-terminal RB8 fragment was replaced by the PstII/NotI-digested VB6 fragment. This resulted in a head-to-tail arrangement of both VB6 nanobodies connected by flexible (Gly_4_Ser_1_)_3_ linker.

For functional characterization in mammalian cells, translational fusions of vimentin-specific sdAbs with eGFP were constructed by introducing BglII/HindIII restriction sites in the target backbone vector (pEGFP-N1, Clontech). BglII and HindIII restriction sites were introduced by PCR using the following set of forward primers comprising a BglII restriction site: 5’-GGG GAG ATC TCC GGC CAT GGC TCA GGT GCA GCT GGT GGA GTC TGG-3’, 5’-GGG GAG TTC TCC GGC CAT GGC TCA GGT GCA GCT GCA GGA GTC TGG-3’, 5’-GGG GAG ATC TCC GGC CAT GGC TCA TGT GCA GCT GCA GGA GTC TGG-3’ in combination with the following set of reverse primers including a HindIII site: 5’-GGG GGA AGC TTC TTG AGG AGA CGG TGA CCT GCA T-3’, 5’-GGG GGA AGC TTC TTG AGG AGA CGG TGA CCT GGG-3’, 5’-GGG GGA AGC TTC TGC TGG AGA CGG TGA CCT GGG T-3’. The PCR products were purified, digested with BglII and HindIII and ligated in the BglII/HindIII sites of the pEGFP-N1 vector. For mammalian expression of vimentin constructs full-length vimentin as well as head, rod and tail domain of vimentin were cloned into pEGFP-C1 vector (Clontech) via the KpnI and BamHI restriction sites. Following PCR primer sets were used to introduce KpnI/BamHI restriction sites: Vim-fwd (5’-AAG GGT ACC TCC ACC AGG TCC GTG TCC-3’); Vim-rod-fwd (5’-AAA GGT ACC AAG AAC ACC CGC ACC AAC GAG-3’); Vim-tail-fwd (5’-AAA GGTA CCA GCA GGA TTT CTC TGC CTC TTC C-3’); Vim-head-rev (5’-AAA GGA TCC TCA GAA CTC GGT GTT GAT GGC-3’); Vim-rod-rev (5’-AAG GGA TCC TCA CTC CTC GCC TTC CAG CAG-3’); Vim-rev (5’-AAG GGA TCC TCA TTC AAG GTC ATC GTG ATG C-3’). The PCR products were purified, digested with KpnI and BamHI and ligated in the KpnI/BamHI sites of the pEGFP-C1 vector. All resulting constructs were sequenced and tested for expression in HEK293T followed by western blot analysis.

**Protein expression and purification.** Expression of vimentin-His_6_ was carried out in *E. coli* BL21(DE3) CodonPlus-RIL (Stratagene) as described previously [^2^](#_ENREF_2). Subsequently, cells were lysed under denaturating conditions (40 mM NaHCO_3_, 1 % SDS, 300 mM NaCl, 20 mM β-mercaptoethanol, pH 9.6) for 1 h at 37 °C, followed by sonication (10 times for 20 s). After centrifugation (15,000 x g, 30 min), the supernatant was diluted 1:4 in binding buffer (2 x phosphate buffered saline (PBS), 0.125 % SDS, 5 mM β-mercaptoethanol, 5 mM imidazole, pH 8.0) and separated by IMAC. For elution binding buffer containing 500 mM imidazole, pH 7.5 was used and buffer exchange of vimentin-His_6_-containing fractions into PBS was carried out with PD-10 Desalting Columns (GE Healthcare). Expression and purification of vimentin-specific sdAbs was carried out as described previously ^[4](#_ENREF_4)^.

**Affinity measurements.** For mammalian expression of GFP-labeled vimentin (GFP-VIM) or GFP, 1 x 10^7^ HEK293T cells each were seeded in p100 culture dishes and transiently transfected with equal amounts of expression vectors encoding for GFP-VIM or eGFP. Transfection efficiency was monitored on the next day by fluorescence microscopy and cells were harvested 48 h after transfection. Cell pellets were lysed as described previously. For determination of the amount of GFP-VIM or GFP in the soluble protein fraction after centrifugation (10 min, 18,000 x g) aliquots (undiluted / 1:10 dilution, 50 µl) were transferred into a 96 well microplate (Nunc). In parallel, serial dilutions of purified GFP ranging from 25 ng – 10 µg were titrated in the same plate generating a standard curve. For protein determination the 96 well plate was subjected to laser scanning using a Typhoon Trio (GE Healthcare Life Sciences, excitation: 488 nm, emission filter settings: 520 nm BP 40). Based on the linear standard curve the amount of fluorescent protein (GFP-VIM, GFP) in the soluble protein fraction was calculated. To estimate the affinity of the VB3 and VB6 nanobodies the BLItz^TM^ (ForteBio) system was used. Purified VB3 and VB6 were immobilized via their C-terminal His6-tag on Anti-Penta-HIS *dip and read* biosensors. Individual VB3 or VB6 coated biosensors were incubated with the soluble protein fraction comprising GFP-VIM or GFP. At least five readings at different GFP-VIM concentrations (9 – 370 nM) were used for VB3 and VB6 and a global fit was applied to calculate binding affinities. As negative control the highest concentration of GFP (370 nM) was used for the binding studies.

**Resazurin assay.** HeLa cells transiently expressing VB3-CB, VB6-CB or eGFP for 72 h were incubated for one hour in cell culture medium containing resazurin (Sigma Aldrich) at a final concentration of 10 µg/ml. Fluorescence was measured with a PHERAstar plate reader (BMG Labtech). Student’s t-test was performed for statistical analysis.

**Wound healing assay.** A549-wt or A549_VB6-CB cells were seeded in 12-well plates and grown to 90 % confluence. Three scratch wounds per well were applied with a 200 µl pipette tip and non-adherent and cells were washed twice with PBS. Fresh medium with or without 5 ng/mL TGF-β was added and cells were cultured for 48 h. Phase contrast images were taken immediately (0 h), 24 h and 48 h after wounding with a Cell Observer SD (Zeiss), 10 x magnification. Open wound areas were determined with the *TScratch automated analysis* software [^5^](#_ENREF_5). For statistical analysis student’s t-test was used.

**Transwell invasion assay** Polycarbonate membrane transwell inserts (8 µM pre size, 0.33 cm^2^, Corning) for 24-well plates were coated with 0.4 mg/mL growth factor reduced Matrigel (BD Biosciences) according to the manufacture’s guidelines. A549-wt or A549_VB6-CB cells were seeded at 50,000 cells per well on Matrigel coated inserts in FCS free medium in presence or absence of TGF-β and 10 % FCS was added to the bottom wells of the chambers. After 24 h and 48 h cells were washed with PBS, fixed with 100 % Methanol for 15 min and stained with crystal violet (1 % crystal violet, 10 % ethanol in H_2_O) for 30 min. Non-invading cells were swiped off and phase contrast images were taken with Cell Observer SD (Zeiss), 10 x magnification. The percentage of invading cells was determined with ImageJ software.

**Quantitative real-time PCR.** A549-wt or A549-VB6-CB cells were stimulated with TGF-β for 0 h, 24 h, 48 h or 72 h. RNA was prepared using the RNeasy Mini Kit (Qiagen). RNA samples were subjected to a DNase digest according to manufacturer’s protocol (RQ1-DNase, Promega) to remove any residual genomic DNA. Using M-MuLV reverse transcriptase (New England Biolabs) and hexanucleotide primers (dN6-primers, Sigma Aldrich) cDNA was synthesized. Real-time PCR analysis was conducted with a StepOne Plus system (Applied Biosystems, Life technologies) using predesigned exon-spanning probes for GAPDH, E-cadherin (*CDH1*), Snail (*SNAI1*) and Slug (*SNAI2*) (Integrated DNA Technologies). Relative mRNA expression levels were determined by the 2(-ΔΔC(T)) method [^6^](#_ENREF_6).

**Supplementary References**

1 Rothbauer, U. *et al.* Targeting and tracing antigens in live cells with fluorescent nanobodies. *Nature methods* **3**, 887-889, doi:10.1038/nmeth953 (2006).

2 Traenkle, B. *et al.* Monitoring interactions and dynamics of endogenous beta-catenin with intracellular nanobodies in living cells. *Molecular & cellular proteomics : MCP*, doi:10.1074/mcp.M114.044016 (2015).

3 Arbabi Ghahroudi, M., Desmyter, A., Wyns, L., Hamers, R. & Muyldermans, S. Selection and identification of single domain antibody fragments from camel heavy-chain antibodies. *FEBS letters* **414**, 521-526 (1997).

4 Rothbauer, U. *et al.* A versatile nanotrap for biochemical and functional studies with fluorescent fusion proteins. *Molecular & cellular proteomics : MCP* **7**, 282-289, doi:10.1074/mcp.M700342-MCP200 (2008).

5 Geback, T., Schulz, M. M., Koumoutsakos, P. & Detmar, M. TScratch: a novel and simple software tool for automated analysis of monolayer wound healing assays. *BioTechniques* **46**, 265-274, doi:10.2144/000113083 (2009).

6 Livak, K. J. & Schmittgen, T. D. Analysis of relative gene expression data using real-time quantitative PCR and the 2(-Delta Delta C(T)) Method. *Methods* **25**, 402-408, doi:10.1006/meth.2001.1262 (2001).
