# Supplementary figures and images for: Real-time analysis of epithelial-mesenchymal transition using fluorescent single-domain antibodies
Source: Sci Rep. 2015 Aug 21;5:13402. doi: 10.1038/srep13402 (PMC4544033; doi:10.1038/srep13402)

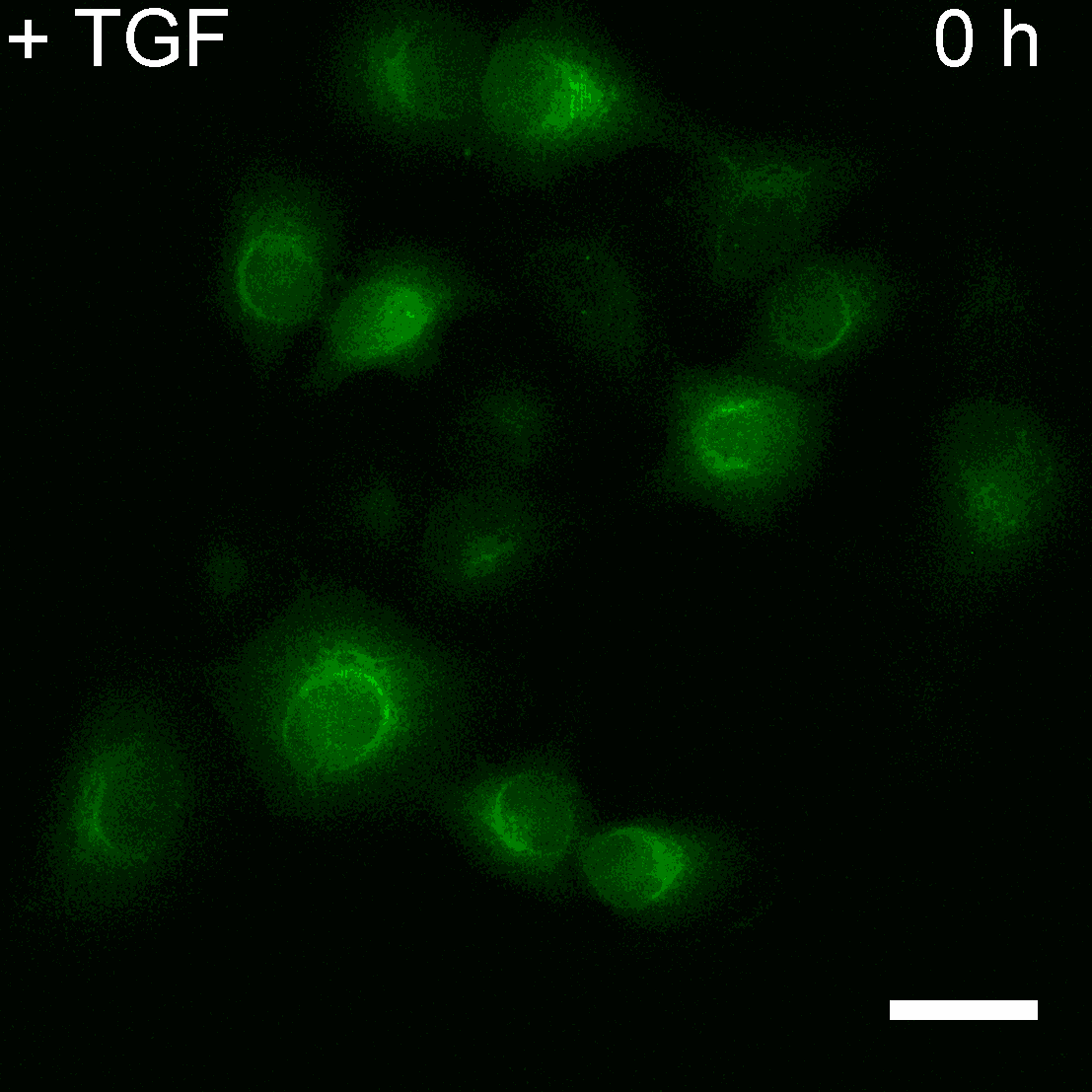

Supplement: Supplementary movie [file srep13402-s2.gif]
